# Cytosolic antibody receptor TRIM21 is required for effective tau immunotherapy in mouse models

**DOI:** 10.1126/science.abn1366

**Published:** 2023-03-30

**Authors:** Aamir S Mukadam, Lauren VC Miller, Annabel E Smith, Marina Vaysburd, Siri A Sakya, Sophie Sanford, Sophie Keeling, Benjamin J Tuck, Taxiarchis Katsinelos, Chris Green, Lise Skov, Sanne S Kaalund, Stian Foss, Keith Mayes, Kevin O’Connell, Mark Wing, Claire Knox, Jessica Banbury, Edward Avezov, James B Rowe, Michel Goedert, Jan Terje Andersen, Leo C James, William A McEwan

**Affiliations:** 1 UK Dementia Research Institute at the University of Cambridge, Hills Road, Cambridge CB2 0AH, UK; 2 MRC Laboratory of Molecular Biology, Francis Crick Avenue, Cambridge CB2 0QH, UK; 3 Department of Immunology, University of Oslo and Oslo University Hospital Rikshospitalet, N-0424 Oslo, Norway; 4 Institute of Clinical Medicine and Department of Pharmacology, University of Oslo and Oslo University Hospital, N-0372 Oslo, Norway; 5 Department of Clinical Neurosciences, University of Cambridge, CB2 0AH; 6 Cambridge University Hospitals NHS Trust, Cambridge, CB2 0SZ

## Abstract

Aggregates of the protein tau are proposed to drive pathogenesis in neurodegenerative diseases. Tau can be targeted using passively transferred antibodies (Abs) but the mechanisms of Ab protection are incompletely understood. Here we used a variety of cell and animal model systems and showed that the cytosolic Ab receptor and E3 ligase TRIM21 (T21) could play a role in Ab protection against tau pathology. Tau:Ab complexes were internalised to the cytosol of neurons, which enabled T21 engagement and protection against seeded aggregation. Ab-mediated protection against tau pathology was lost in mice lacking T21. Thus, the cytosolic compartment provides a site of immunotherapeutic protection, which may help in the design of Ab-based therapies in neurodegenerative disease.

## Main text

Several neurodegenerative diseases are characterised by the progressive accumulation of cytosolic assemblies of hyperphosphorylated tau (1). Extracellular tau assemblies are taken up into recipient cells following interactions with surface heparan sulphate proteoglycans and low density lipoprotein receptor-related protein 1 (LRP1) (2–5) and can induce seeded aggregation of native tau pools (6). Passive transfer of antibodies (Abs) against tau can reduce tau pathology in animal models and is under investigation as a disease-modifying treatment in humans (7–11). The mechanisms of this protection remain uncertain with roles for microglial internalisation using cell surface Ab receptors, FcγRs (12–14), blocking of seed entry to cells (13, 15, 16), and endolysosomal degradation (17–19) suggested as potential modes (20). The cytosolic Ab receptor and E3 ubiquitin ligase, TRIM21 (T21), engages Ab-bound particles inside the cell and mounts a potent degradation response at the proteasome (21–23). In cell-based assays, the introduction of Abs to cells can induce the T21-dependent selective degradation of numerous cellular proteins including tau (24–26). However, the contribution of T21 to immunotherapeutic protection against tau pathology remains undetermined.

## Tau assemblies enter neurons in complex with Ab to contact T21

**Figure 1:**
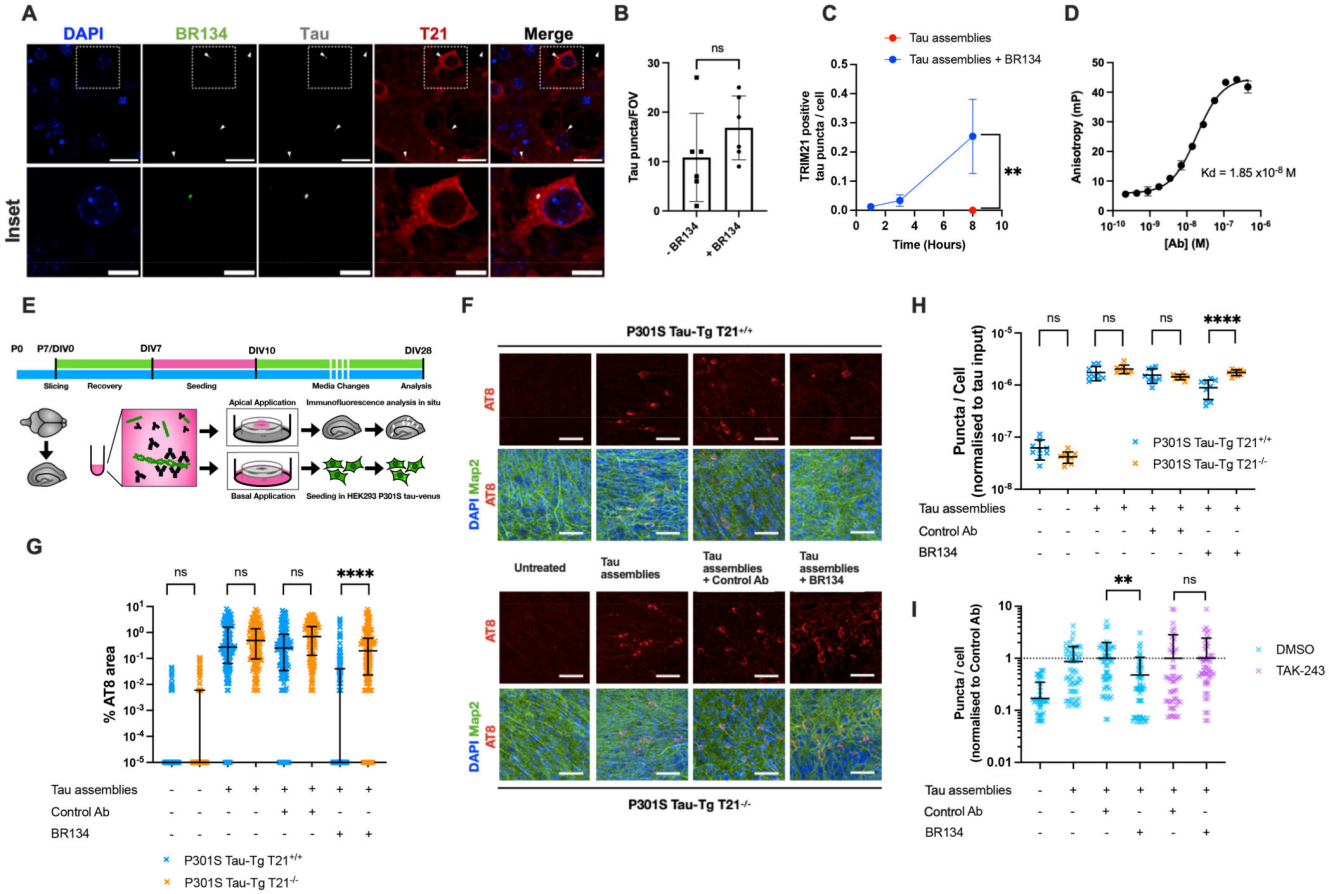
Mechanisms of Ab protection in neuronal cultures. A) Confocal immunofluorescence microscope images of mouse primary neurons expressing mCherry-T21 treated with tau assemblies in complex with tau C-terminus specific rabbit polyclonal Ab, BR134. Arrows indicate intracellular Ab:tau assembly complexes, the majority of which were found to colocalise with T21. Scale bar 25 μm, inset scale bar 10 μm. B) Number of tau assemblies detectable within neurons 8 h after their addition in the presence or absence of BR134. C) Number of intracellular tau puncta that colocalise with T21 in the presence or absence of BR134. D) Fluorescence anisotropy of Alexa488-labelled mouse T21 PRYSPRY domain in the presence of indicated concentration of BR134. E) Diagram of organotypic hippocampal slice culture (OHSC) model of seeded tau aggregation. Tau assemblies are pre-treated with Abs and provided to hippocampal slices prepared from P301S Tau-Tg animals on day in vitro (DIV) 7. OHSCs are fixed for immunofluorescence analysis of tau pathology (AT8) on DIV28 or lysed and examined for levels of tau seeding in HEK293 P301S tau-venus reporter cells. F) Representative immunofluorescence images for AT8-reactive tau structures in OHSCs from P301S Tau-Tg T21^+/+^ and T21^-/-^ backgrounds challenged with tau assemblies that were untreated or incubated with control Ab 9C12 or BR134. Map2 staining reveals neuronal architecture. Scale bar 50 μm. G) Levels of AT8 reactivity in OHSCs following treatment with tau assemblies in the absence of Abs, or after pre-incubation with the indicated Ab. H) Levels of tau seeds present within OHSCs 3 weeks following treatment with indicated tau and Ab complexes. Levels were determined by applying OHSC homogenates to HEK293 cells expressing P301S tau-venus. I) Levels of AT8-reactive tau structures in primary neurons derived from P301S Tau-Tg mice challenged with tau assemblies that were untreated or incubated with the indicated Ab in the presence of DMSO or E1 inhibitor TAK-243. Data normalised to control antibody. Median and interquartile range indicated. B-C) Mean +/- sd from N=6 randomly selected fields of view; D) Mean +/- sd from N=2 repeats. G) Points represent 100x100 μm sections from images of OHSCs prepared from N=6 mice with median +/- interquartile range. H) Each point represents seeding from pooled OHSC homogenates derived from N=3 mice with median +/- interquartile range. I) Points represent values from individual fields of view from N=3 independent repeats with mean +/- sd. B, C) Mann-Whitney test; G, I) Kruskal-Wallis test with Dunn’s correction for multiple comparisons; H) one-way ANOVA with Tukey’s correction for multiple comparisons; **, P<0.01; ****, P<0.0001.

To investigate whether seeded aggregation of tau may be intercepted by T21, we first asked whether Abs could be taken into neurons in complex with tau assemblies. We incubated recombinant heparin-assembled tau with BR134, a rabbit polyclonal Ab raised against the C-terminus of tau (27) that can neutralise seeded tau aggregation in human cell lines (25). After 8 h, tau assemblies were observed within neurons irrespective of whether Ab was present, indicating that BR134 did not prevent their uptake (Fig 1A,B). Notably, where BR134 was present, these intracellular tau assemblies colocalised with T21, which resides in the cytosol. The number of intracellular T21-positive tau assemblies increased over the course of 8 hours, consistent with the entry dynamics of tau to the cytosol of neurons (28) (Fig 1C; Fig S2). Dimers of T21 bind Abs via interactions between the T21 PRYSPRY domain and the Ab Fc region (29). We confirmed this interaction in the context of BR134 and mouse T21 PRYSPRY domain using fluorescence anisotropy and observed a monomer dissociation constant (Kd) of 19 nM (Fig 1D). Thus tau assemblies can enter the cytosol of neurons in complex with antibodies and recruit T21 via a high-affinity interaction between the Ab Fc region and the T21 PRYSPRY domain.

## T21 and Abs lead to functional inactivation of tau seeding behaviour

We next asked whether T21 contributes to the neutralisation of seeded tau aggregation. We used an organotypic hippocampal slice culture (OHSCs) model of seeding (30). Slices were prepared from transgenic mice expressing human tau with frontotemporal lobar degeneration (FTLD) associated mutation P301S (P301S Tau-Tg) and cultured at the air-liquid interface (Fig 1E). We also prepared slices from a T21-deficient mouse line (31) on the same tau background (32) (P301S Tau-Tg T21^-/-^). OHSCs derived from both genotypes retained normal representation of the major cell types of the CNS and OHSCs from T21^-/-^ animals did not express detectable T21 by immunoblot (Fig S3). OHSCs maintained tau in a native state over 8 weeks in culture and the genotypes displayed similar levels of seeded aggregation in response to heparin assembled tau (Fig S3). However, there was a substantial difference in the observed levels of neutralisation by BR134 between the genotypes (Fig 1F,G). BR134 reduced seeding by >90% in T21^+/+^ OHSCs compared to control Ab. However, genetic deletion of T21 almost completely abolished the ability of BR134 to prevent seeded aggregation. We next asked whether the activity of Abs and T21 could inhibit the formation of seed-competent tau species which occurs as a result of seeded aggregation in the OHSCs. We treated OHSCs with recombinant heparin tau assemblies in the presence and absence of BR134 as above. OHSC homogenates were examined 3 weeks later for the levels of seed-competent species on a sensitive reporter cell line (HEK293 P301S tau-venus (25)). Untreated OHSCs contained only low levels of tau seeds, whereas those treated with tau assemblies induced substantial levels of seeded aggregation (Fig 1H). We observed a significant reduction in tau seeds in response to treatment with BR134 when compared to control Ab, but only when T21 was present. In P301S Tau-Tg T21^-/-^ OHSCs, Abs were unable to reduce the number of seeds that were produced. T21 promotes virus neutralisation via its E3 ligase activity, which stimulates degradation with the involvement of the ubiquitin-proteasome system (UPS) (21). To test whether this activity was required for the neutralisation of seeding, we used TAK-243, an inhibitor of UBA1, an E1 ubiquitin activating enzyme (33), which prevented poly-ubiquitination in primary neurons (Fig S4). Neutralisation of tau seeding was no longer observed when the inhibitor was applied (Fig 1I, Fig S4). Thus, Abs recruit T21 to internalised tau assemblies and inhibit the formation of new seed-competent tau assemblies and reduce levels of hyperphosphorylated tau inclusions.

## Comparison of T21 with classical Fc receptors in organotypic slice culture

**Figure 2:**
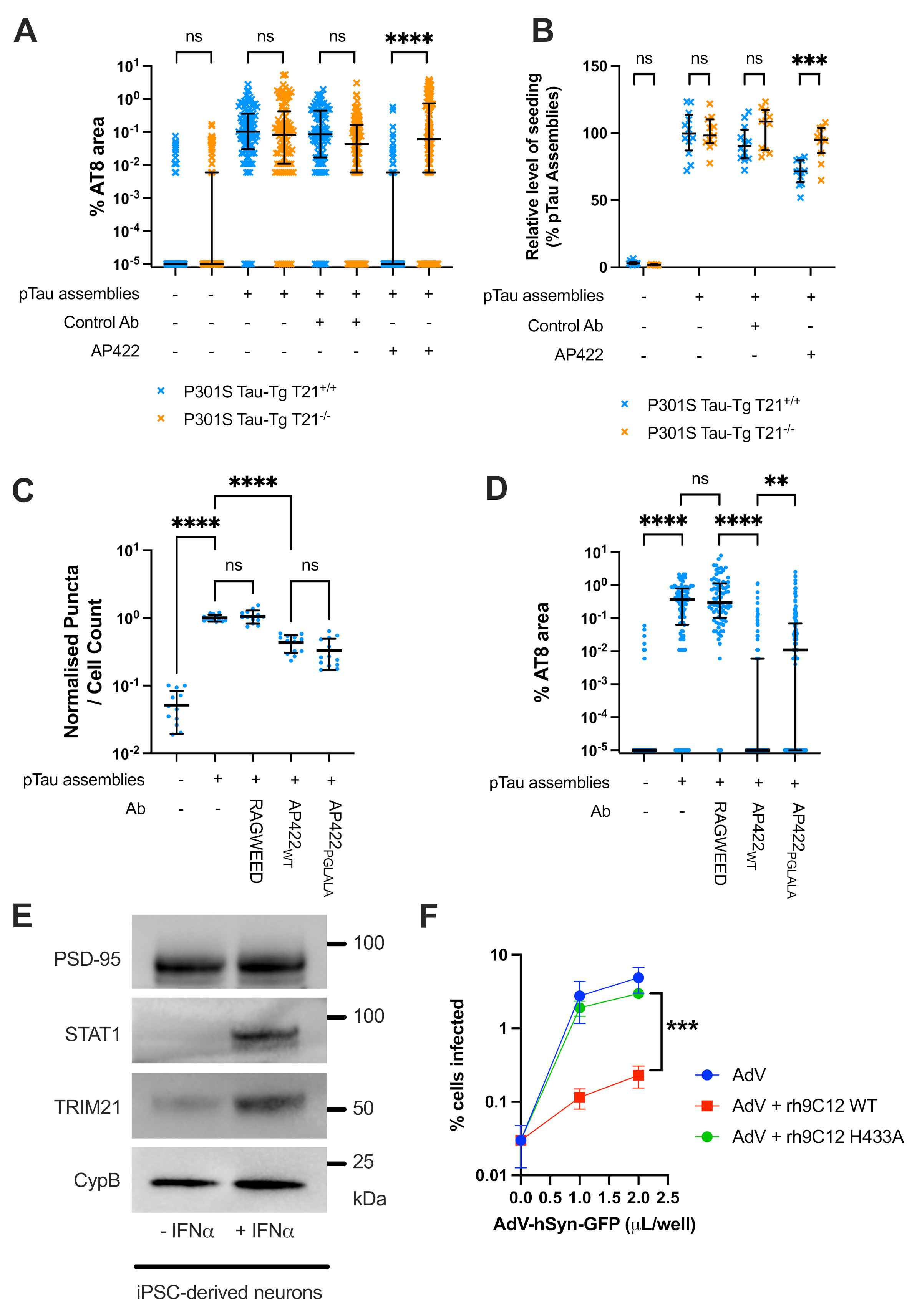
FcγR contributions in OHSCs and T21 in human iPSC-derived neurons. A) Levels of AT8 staining in P301S Tau-Tg T21^+/+^ and T21^-/-^ OHSCs treated with phospho-tau assemblies in the presence of AP422, a mouse IgG1 which binds to tau phosphorylated at S422, or isotype matched anti-adenovirus control, 9C12. B) Levels of seeding observed in extracts prepared from OHSCs treated with the indicated tau assemblies and Abs. C) Levels of seeded aggregation in HEK293 cells treated with 1 nM phospho-tau assemblies in the presence of indicated Abs. D) Levels of AT8 staining in OHSCs treated with phospho-tau assemblies that were incubated with the indicated recombinant Abs. Ragweed, anti-ragweed pollen control; AP422_WT_, mouse IgG2a; AP422_PGLALA_, mouse IgG2a with the PGLALA mutations that prevent FcγR interaction. E) Immunoblots for T21, synaptic marker PSD-95, IFN-stimulated gene STAT-1 and loading control CypB in human iPSC derived neurons in the presence and absence of IFNα. F) Levels of adenovirus type 5 infection in human iPSC derived neurons in the absence of Ab or in the presence of recombinant anti-AdV 9C12 expressed with human IgG1 Fc. Wildtype Fc or Fc bearing H433A which prevents interaction with TRIM21 was used. A-D) Median and interquartile range shown. A,D) Points represent 100x100 μm sections from images of OHSCs prepared from N=6 mice. B) Each point represents seeding from pooled OHSC homogenates derived from N=3 mice. C) N=3 biological replicates, points represent technical replicates. A, D) Kruskal-Wallis test with Dunn’s correction for multiple comparisons. B,C) One-way ANOVA with Tukey’s correction for multiple comparisons. F) Mean and standard deviation; N=3 independent replicates; unpaired t-test. **, P<0.01; ***, P<0.001; ****, P<0.0001.

Abs can mediate extracellular protection against tau by promoting uptake to microglia via interactions with FcγRs (12, 14). We thus examined the contribution of FcγR interaction in preventing seeded aggregation in OHSCs. We used a mouse monoclonal Ab, AP422, which binds to tau phosphorylated at S422 (34) and detects tau prepared from Alzheimer’s disease, corticobasal degeneration and progressive supranuclear palsy brains (Fig S5). We used kinase ERK2 to phosphorylate recombinant tau, which generated the AP422 epitope (Fig S6). Like with BR134, AP422 protected against seeding and generation of seed-competent species in OHSC, both of which were dependent on T21 (Fig 2A,B; Fig S5). To enable reverse genetic mutagenesis of Ab Fc region, AP422 was cloned and expressed as recombinant mouse IgG2a (rAP422). We verified that specificity for phospho-tau was maintained (Fig S6) and introduced the Fc amino acid substitutions P329G, L234A and L235A (PGLALA), which abrogate FcγR interactions (35, 36). ELISA confirmed that the PGLALA substitutions ablated interactions with mouse FcγRI, FcγRIIB, FcγRIII and FcγRIV but maintained T21 and FcRn interactions (Fig S6). As a control, we used recombinant mouse IgG2a against ragweed pollen. As expected, in HEK293 cells, which do not express significant levels of FcγRs (25), there was no difference between neutralisation with rAP422-WT versus rAP422-PGLALA (Fig 2C). In OHSCs, where microglia are present, rAP422-PGLALA was able to exert similar levels of protection as unmodified rAP422-WT, with only a minor portion of its activity being lost (Fig 2D, Fig S6). This contrasts with T21 knockout where neutralisation was almost entirely lost. Thus, intracellular neutralisation via T21 is primarily responsible for protection against tau seeding by AP422 in ex vivo brain slice cultures.

## T21 is present and functional in human iPSC-derived neurons

An important consideration for tau immunotherapy in neurodegenerative disease is the level and activity of T21 in human neurons, the major site of tau expression and aggregation in Alzheimer’s disease and many other tauopathies. We used human iPSC-derived neurons to examine whether T21 is available for Ab-mediated degradation in this setting. Immunoblot confirmed that T21 is expressed in human iPSC-derived neurons, and is upregulated by treatment with IFNα, a cytokine known to regulate T21 expression (Fig 2E) (21, 37). We used neutralisation of an adenovirus type 5 vector expressing GFP under the control of a neuron-specific synapsin promoter (AdV) to determine T21 activity. Treatment of AdV with the anti-hexon mouse monoclonal Ab 9C12 neutralises infection in a T21-dependent manner that can be reversed by the Fc amino acid substitution H433A at the T21 binding interface (22). Using a chimeric mouse-human variant of 9C12 with human Fc region (rh9C12) (38), we observed potent neutralization of infection of AdV infection in human neurons (Fig 2F). However, rh9C12 with point mutation H433A was almost completely unable to neutralize infection. Thus, T21 is expressed and active in human neurons and the T21 pathway is available for engagement by immunotherapy in this cell type.

## Contribution of T21 to in vivo protection during tau immunotherapy

**Figure 3:**
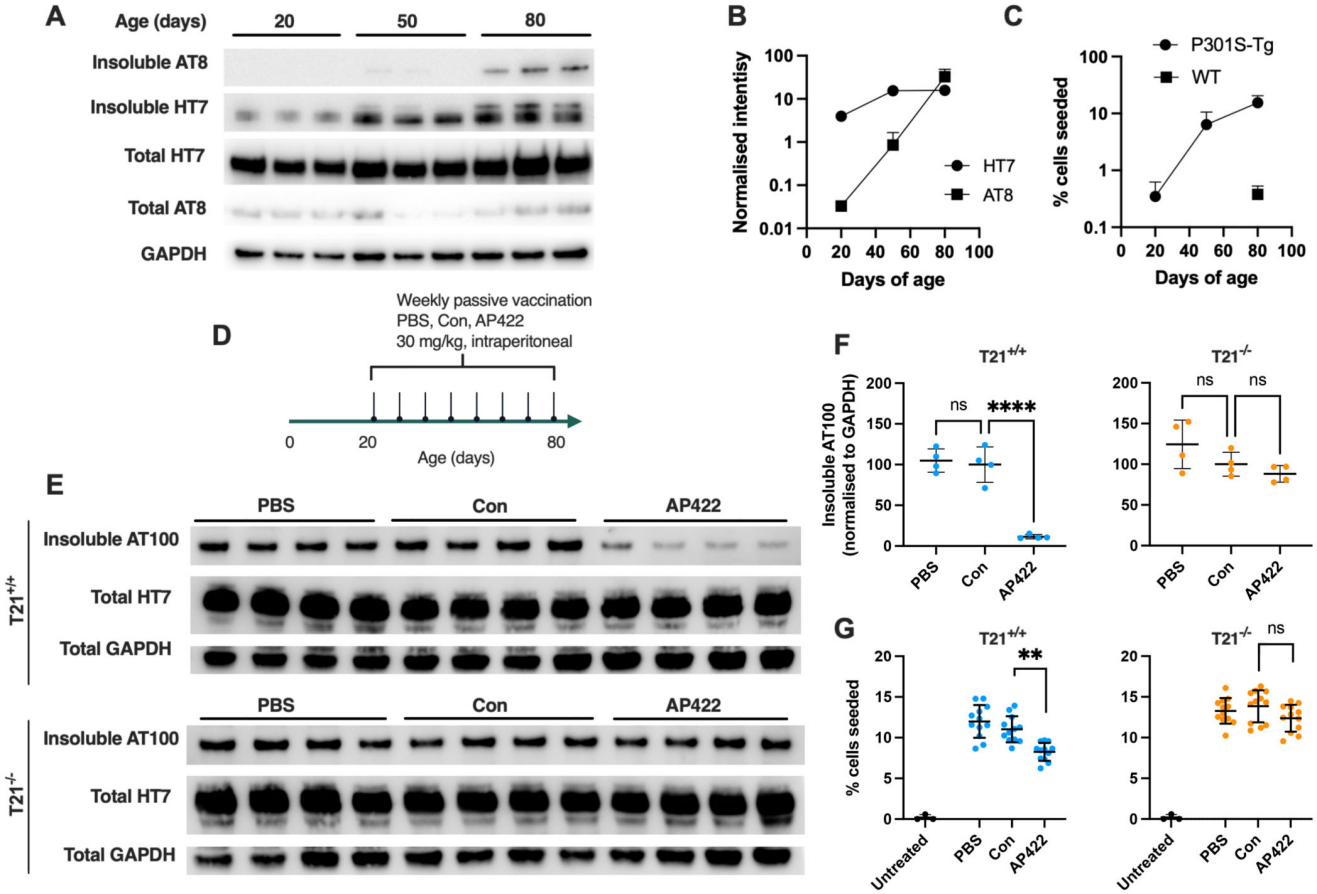
T21 is required for immunotherapeutic protection against incipient tau pathology. A) Immunoblot analysis of total homogenate and sarkosyl insoluble fractions prepared from the lumbar spinal column of P301S Tau-Tg mice at postnatal day 20, 50 and 80. Lanes represent individual animals. B) Quantification of tau in sarkosyl insoluble fractions using Abs AT8 and HT7, normalised to GAPDH. C) Levels of seeding in HEK293 P301S tau-venus cells treated with the same sarkosyl insoluble fractions, or with insoluble fractions from wildtype mice. D) Timeline of antibody treatment with mock (PBS), anti-adenovirus 9C12 (Con) or anti-pS422 tau (AP422) by weekly i.p. injection between ages 20-80 days. E) Immunoblot analysis of total and sarkosyl insoluble fractions of spines from treated mice. Each lane represents an individual mouse. F) Quantification of AT100 levels normalised to GAPDH from E). G) Levels of seed competent tau present in spine sarkosyl insoluble fraction derived from mice treated with the indicated Ab, points represent average seeding in multiple wells from N=4 mice. F) Mean +/- sd and one-way ANOVA with Dunnett’s multiple comparison test. G) Mean +/- sd and nested one-way ANOVA; **, P<0.01; ***, P<0.001.

We next investigated the role of T21 in a transgenic animal model of tau pathology. In P301S-Tg mice, incipient tau pathology can be detected by immunoreactivity to phospho-tau in lumbar spinal sections from 1 month followed by amplification of signal until 7 months (39). Sarkosyl insoluble tau and seed-competent species increased between 20 and 80 days of age (Fig 3A-C). No seed-competent species were detected in non-transgenic C57BL/6 mouse spines (Fig 3C), indicating that seeding activity arises from transgenic tau. We therefore asked whether Abs could reduce incipient tau pathology by passive Ab transfer into P301S Tau-Tg T21^+/+^ and P301S Tau-Tg T21^-/-^ mice. Mice were treated with AP422, control Ab 9C12 (Con) or buffer only (PBS) by weekly intraperitoneal (i.p.) injection (Fig 3D). Immunoblot revealed a greater than 85% reduction in insoluble tau levels following AP422 treatment in T21^+/+^ animals (Fig 3E,F). However, no Ab protection was observed in T21^-/-^ animals or when control Ab was used. We further examined levels of seed-competent tau species in these preparations and observed a significant T21-dependent reduction following AP422 treatment (Fig 3G). Of note, the protection against sarkosyl insoluble tau accumulation was of greater magnitude than the reduction in the generation of new seed competent species. This is of interest because T21 is activated by a stoichiometric threshold of Abs (40). Given that tau seeds can be of low stoichiometric value, potentially even monomers (41, 42), our findings suggest that fibrillar tau may represent a better substrate for T21 degradation than small tau assemblies. In summary, Abs can reduce levels of incipient tau aggregation in the mouse brain in a manner that requires T21.

## In vivo requirement of T21 during long-term immunotherapy

**Figure 4:**
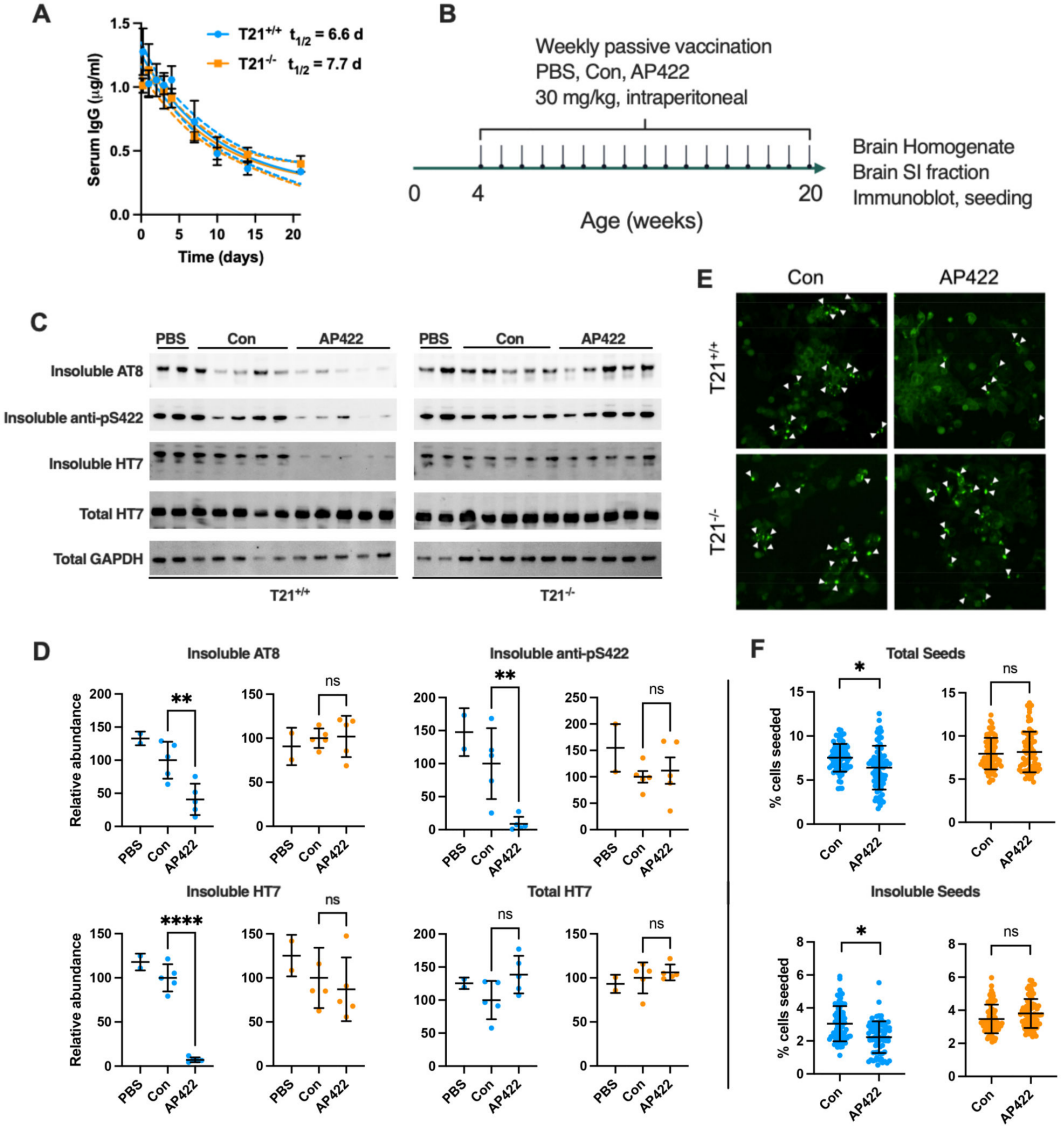
Long-term immunotherapy potentiates T21-dependent protection against tau pathology. A) Serum concentration of biotinylated Abs at indicated time following injection of 30 mg/kg to N=3 P301S-Tg mice that were either T21^+/+^ or T21^-/-^. B) Cartoon depicting timeline of antibody treatment. Mice were treated with mock i.p. injection (PBS), control Ab 9C12 (Con) or anti-tau (AP422) for 17 weeks. Total homogenate and sarkosyl insoluble fractions were prepared for immunoblot and quantification of seed-competent species. C) Immunoblot of total and sarkosyl insoluble fractions from brain hemispheres from P301S-Tg mice that were either T21^+/+^ or T21^-/-^. Samples were probed with either pan-tau monoclonal antibody HT7, or phospho-specific tau Abs AT8 and anti-pS422. Each lane represents a single mouse. D) Quantification of HT7, AT8, and pS422 levels normalised to GAPDH using the same samples as C). E) Images of HEK293 cells expressing P301S tau-venus treated with diluted sarkosyl insoluble fractions from brains treated with the indicated Ab or PBS. F) Quantification of seed competent tau present in brain homogenates and sarkosyl insoluble fractions derived from mice treated with the indicated Ab. Points represent seeding in individual images from N=5 mice. A) Mean +/- sd; one-phase decay curves compared using extra sum-of-squares F-test, not significant. D-F) Mean +/- sd. D) one-way ANOVA with Dunnett’s test for multiple comparisons; F) nested t-test for individual mice; *, P<0.05; **, P<0.01; ****, P<0.0001.

We further sought to establish the involvement of T21 in a chronic Ab treatment regimen in adult mice. We first examined the persistence of biotinylated Abs in circulation and observed similar half-lives (~ 7 days) in the P301S Tau-Tg versus P301S Tau-Tg T21^-/-^ mice (Fig 4A). This suggests that T21 is not involved in determining Ab persistence. We therefore proceeded to treat both genotypes with PBS, 9C12 (Con) or AP422 for 17 weeks by weekly administration of Abs to the periphery (Fig 4B). AP422 conferred potent protection against total sarkosyl-insoluble tau (HT7) and against hyperphosphorylated sarkosyl-insoluble tau species detected by Abs AT8 and anti-pS422, which detects the phospho-epitope targeted by AP422 (Fig 4C,D). A reduction in pS422-postive cell bodies was also observed by immunofluorescence microscopy (Fig S7). However, in T21-deficient mice, AP422 was again unable to protect against tau pathology. We quantified the number of seed-competent species in brain homogenate and sarkosyl-insoluble fractions using HEK293 P301S tau-venus cells. AP422 was able to reduce levels of seeds in both fractions compared to the control 9C12, but only when T21 was expressed (Fig 4E,F). Thus, T21 is required for protection against tau pathology during chronic immunotherapy in mice.

## Discussion

In this study we have demonstrated that immunotherapy against tau relies predominantly on the intracellular Ab receptor T21. Mice lacking expression of T21 were refractory to passive immunotherapy at both an early stage of tau pathogenesis, and during prolonged Ab treatment. In T21^-/-^ animals, Abs were unable to reduce level of insoluble tau in the brain and failed to reduce the generation of seed competent species. In ex vivo slice culture experiments, we observed that seeded aggregation was susceptible to Ab neutralisation in a manner that relied largely on T21. We found that other effector mechanisms mediated by cell surface FcγRs, expressed widely on microglia in the brain, were less effective than T21 in our models. We found that tau:Ab complexes were internalised to neurons, and were recognised by T21 in the cytosol. Our findings are therefore consistent with Abs binding to extracellular tau species before uptake of the tau:Ab complexes to cells. Subsequent binding of T21 to Abs neutralised seeding activity in a manner that required active ubiquitination machinery. Our findings have implications for immunotherapy as a putative treatment in neurodegenerative disease. Indeed, human iPSC-derived neurons were found to express regulatable and functional T21. Finally, it is possible that proteins other than tau with similar prion-like behaviour such as α-synuclein and TAR DNA-binding protein (TDP)-43 may be similarly susceptible to T21-mediated neutralisation.

## Supplementary Material

Supplementary Materials

## Data Availability

All data are available in the manuscript or the supplementary material.

## One sentence summary

Passive transfer of antibodies targeting tau can reduce tau pathology via the cytosolic antibody receptor and E3 ligase TRIM21.
