# Supplementary material for: Cytosolic antibody receptor TRIM21 is required for effective tau immunotherapy in mouse models
Source: Science. Author manuscript; Available in PMC 2023 May 9. (PMC7614512; doi:10.1126/science.abn1366)
Supplement: Supplementary Materials [file EMS174787-supplement-Supplementary_Materials.pdf]

Supplementary Materials for

Cytosolic antibody receptor TRIM21 is required for effective tau immunotherapy in mouse models

Aamir S Mukadam[1,5 †], Lauren VC Miller[1,5 *], Annabel E Smith[1,5*], Marina Vaysburd[2], Siri A Sakya[3,4], Sophie Sanford[1,5], Sophie Keeling[1,5], Benjamin J Tuck[1,5], Taxiarchis Katsinelos[1,5], Chris Green[1,5], Lise Skov[1,5], Sanne S Kaalund[5], Stian Foss[3,4], Keith Mayes[2], Kevin O'Connell[2], Mark Wing[2], Claire Knox[2], Jessica Banbury[2], Edward Avezov[1,5], James B Rowe[5,6], Michel Goedert[2], Jan Terje Andersen[3,4], Leo C James[2], William A McEwan[1,5 †].
Correspondence to: asm64@cam.ac.uk, wm305@cam.ac.uk

This PDF file includes:

Materials and Methods
Figs. S1 to S7

Materials and Methods

Cell culture

HEK293 cells were maintained in complete DMEM with 10% vol/vol fetal calf serum (FCS), 100 U/ml penicillin and 100 µg/ml streptomycin at 37 °C in a 5% CO_2 humid atmosphere. Hybridoma cells were cultured in OptiMEM supplemented with 2% FCS in cell factory systems (Thermo Fisher Scientific) and the supernatant was harvested twice-weekly. Supernatant was filtered through a 0.22 µm 500 ml filter units (Thermo Fisher Scientific) and stored at 4 °C before purification.

Mice and in vivo immunotherapy

All animal work was licensed under the UK Animals (Scientific Procedures) Act 1986 and was approved by the Medical Research Council Animal Welfare and Ethical Review Body. C57BL/6 *Trim21*$^{-/-}$ mice (MGI: 3849316) were obtained from Jackson Laboratories. P301S tau-transgenic mice (*31*) (MGI: 3778191), which express 0N4R tau under the control of a Thy1 promoter, were extensively backcrossed to C57CL/6. Through the course of the study, animals were weighed and observed twice daily for clinical signs including subdued behaviour, pilo-erection, hunched posture, ataxia and paresis. Animals that displayed clinical signs that did not improve within a 6-hour period were sacrificed. 20 day old P301S tau transgenic mice and age-matched *Trim21*$^{-/-}$ P301S tau transgenic mice were injected weekly (intraperitoneal or i.p.) for 60 days with either 30 mg/kg of mAb AP422, 30 mg/kg of anti-AdV hexon antibody 9C12, or treated with PBS. Post exsanguination, the lumbar regions of the spinal cords were harvested and snap frozen in liquid nitrogen for downstream biochemical and tau seeding analyses. Alternatively, mice were injected weekly intraperitoneally for 17 weeks with either 30 mg/kg of AP422, 30 mg/kg of 9C12 or PBS. Post exsanguination, whole brains including the brainstem and cerebellum were snap frozen for downstream biochemical analyses.

Organotypic hippocampal slice cultures

Organotypic hippocampal slice cultures were prepared and cultured as described previously (*29*). Brains from P6-P8 pups were rapidly removed and kept in ice-cold Slicing Medium (EBSS + 25 mM HEPES) on ice. All equipment was kept ice-cold. Brains were bisected along the midline and the cerebellum was removed using a sterile scalpel. The medial cut surface of the brain was adhered to the stage of a Leica VT1200S Vibratome using cyanoacrylate (Loctite Super Glue) and the vibratome stage was submerged in ice-cold Slicing Medium. Hemispheres were arranged such that the vibratome blade sliced in a rostral to caudal direction. Sagittal slices of 300 μm thickness were prepared and the hippocampus was sub-dissected using sterile needles. 5-8 slices were typically collected per hemisphere. Hippocampal slices were transferred to 15 ml tubes filled with ice-cold Slicing Medium using sterile plastic pipettes with the ends cut off. Slices were then transferred onto sterile 0.4 μm pore membranes (Millipore PICM0RG50) in 6-well plates pre-filled with 1 ml pre-warmed Culture Medium (50% MEM with GlutaMAX, 18% EBSS, 6% EBSS + D-Glucose, 1% Penicillin–Streptomycin, 0.06% nystatin and 25% Horse Serum) and incubated at 37 °C in a humid atmosphere with 5% CO_2. Three slices were typically maintained per well. 24 h after plating 100% media was exchanged and thereafter a 50% media exchange was carried out twice per week for up to 8 weeks.

Seeding neutralisation in OHSCs

OHSCs were prepared and cultured to DIV7. Tau assemblies were mixed with antibodies or buffer only at 1:5 (Tau:Ab) for 1 h at room temperature, before dilution to 300 nM tau in Culture Medium. For analysis by IF, tau assemblies were diluted in a volume of 20 μl Culture Medium, and applied to the apical surface of each OHSC. For analysis by secondary seeding, due to the possibility of the applied tau aggregates adhering to the surface of the OHSC, tau:Ab complexes were added to the media beneath the membrane in a volume of 1 ml, at a final concentration of 300 nM tau. The basal and apical application methods were previously shown to give comparable levels of seeded aggregation (*29*). After 3 days, a 100% media change was performed to remove the tau aggregates. Slices were maintained as described above, for three weeks post seeding, to DIV28. For analysis by IF, OHSCs were fixed in 4% PFA for 20 minutes. Slices

were washed in PBS before the membrane was cut out of the insert. The membrane was used to transfer the slices into 24 well plates, where they were stained with MAP2 (ab5392, Abcam), AT8 (MN1020, Thermo Fisher), tau (A0024, DAKO) antibodies and the nuclei were stained with Hoechst. The membranes were then mounted slice side up, on glass slides, with ProLong Diamond Antifade Mountant (Invitrogen, P36961), and a cover slip was placed on top. OHSCs were imaged using a SP8 Lightning Confocal microscope (Leica). Regions of the highest seeding density were imaged, rather than anatomical regions within the hippocampus, due to variability between slices. Images were analysed in 100 µm x 100 µm sections, using a custom-made macro in Fiji-ImageJ (*44)*, which determined AT8 positive area above a defined threshold in max intensity projections. For analysing the level of seed competent species present in the OHSCs, 3 OHSCs were pooled per extraction, and 3 extractions were completed per condition from different mice (N=3 mice, n=9 OHSCs per condition). OHSCs were seeded +/- antibody at DIV7 beneath the culture membrane and cultured to DIV28. A scalpel was used to remove the OHSCs from the culture membrane and place them in an Eppendorf tube (3 OHSCs per tube). 60 µl of PBS with 1x protease and phosphatase inhibitors was added to each tube. The Eppendorf's were freeze-thawed 5x on dry ice to lyse the cells. To evaluate secondary seeding, 2 µl of whole lysate was mixed with 0.5 µl lipofectamine 2000 in a volume of 50 µl OptiMEM, per well of a 96 well plate. The mixture was added to HEK293 reporter cells, as previously described *(30)*. Each lysate was applied in triplicate. The rest of the seeding assay was carried out as described in 'Seeding Assay in HEK293'.

Extraction of tau assemblies from mouse brains and spinal cords

Insoluble tau was extracted from brain and spinal cords using the sarkosyl extraction protocol (*3*) with modifications as previously described (*29*). Briefly, tissues were homogenised in ice-cold H-Buffer (10 mM Tris pH 7.4, 1 mM EGTA, 0.8 M NaCl, 10% sucrose, protease and phosphatase inhibitors (Halt™ Protease and Phosphatase Inhibitor Cocktail)) using the VelociRuptor V2 Microtube Homogeniser (Scientific Laboratory Supplies). The homogenates were spun for 20 min at 20,000× *g* and supernatant collected. Sarskosyl was added to a final concentration of 1% to the supernatants and

incubated for 1 h at 37 °C. Supernatants were then centrifuged at 100,000× *g* at 4 °C for 1 h. The resulting pellet was resuspended in 0.2 volumes (weight of tissue) of TBS and sonicated for 15 s in a water-bath sonicator before storage at -80 °C for immunoblotting and tau seeding assays.

Tau pathology analysis by immunofluorescence

Mouse brain hemispheres were immersion-fixed for 48 h in 4% paraformaldehyde with PBS, dehydrated and cryoprotected in 30% sucrose in PBS for an additional 72h. The hemisphere was frozen rapidly in 5-methyl butane and 25 µm-thick sagittal sections were cut serially through the entire hemisphere. Three sections per mouse brain (at least 300 µm apart) were stained for semi-quantitative immunohistochemistry. Briefly, brain sections were permeabilised using 0.3% Triton X-100 in PBS, and blocked in 3% horse serum in the same solution (1h, RT). Sections were incubated overnight with AT8-Biotin (ThermoFisher, MN1020B) in permeabilisation buffer at 4°C. Sections were washed in permeabilisation buffer and incubated with Streptavidin conjugated AlexaFluor647 (2h, RT) and Hoechst 33342 in PBS (10ug/mL, ThermoFisher, 15 min). After mounting, sections were evaluated using Nikon Ti2 microscope and pathology quantified blinded. Quantification of AT8-immunopositive cell bodies in the cerebral cortex was manually calculated.

Primary neuron culture

Brains were removed from the heads of P0 or P1 mice and pooled cortex and hippocampal neuronal cultures were prepared as previously described (*27, 43*). Hippocampi and cortices were dissected in ice cold Hibernate-A (Gibco, A1247501) and the meninges removed. The tissue was pooled in a 15 mL conical tube and washed twice with ice-cold Hibernate-A. Tissue was incubated with a final concentration of 0.25% trypsin (Gibco, 15090-046), at 37 °C for 20 minutes. During this period, a cotton-plugged glass Pasteur pipette (Merck Life Science, S6143) was fire-polished. Following trypsinisation, 500 µl 1% (w/v) DNAse I (Sigma-Aldrich, DN25) was added to the tissue and incubated at room temperature for 5

minutes. The tissue was washed twice with room-temperature Hibernate-A, followed by two washes with neuron plating medium (PM) containing Neurobasal Plus (Gibco, A3582901), 1 mM GlutaMAX (Gibco, 15050061), 1 % penicillin-streptomycin (Invitrogen, 15140122), 10 % horse serum (Invitrogen, 26050070), and 1 X B-27 Plus supplement (Gibco, A352801). After washing, 2.5 ml PM was added to the tissue, and the tissue applied to a 60 mm dish. The tissue was triturated using the glass pipette. A further 8 mL of PM was added to the dish, and the cell suspension strained through a 70 μm cell strainer. Live cells were counted via trypan blue staining using the Countess II automated cell counter (Invitrogen). For neutralisation experiments, 30,000 cells/well were seeded into black 96-well plates (Greiner Bio-One, 655090), coated with poly-L-lysine (RnD Systems, 3438-100-01). For confocal imaging, 90,000 cells/well were seeded into poly-L-lysine coated 8-well chamber slides (Thermo Scientific, 154534). After 4 h, all media was removed and replaced with maintenance media (MM) (PM without serum). All primary cultures were maintained in a humidified tissue culture incubator at 37 °C with 5 % CO_2.

Microscopy of tau and T21 colocalisation

Primary neurons were prepared from C57BL/6 $T21^{-/-}$ mice in poly-L-lysine coated 8-well chamber slides (Thermo Scientific, 154534). On DIV 2, primary neurons were infected with AAV1/2 hSyn mCherry-T21 to reconstitute expression. On DIV 7, recombinant tau assemblies and BR134 antibody or buffer were incubated together for 1 hour at room temperature at a 1:5 molar ratio (Tau:Ab). Tau:Ab incubations were diluted in MM and applied to neurons at varying time points at a final concentration of 10 nM tau assemblies and 50 nM BR134. To reduce extracellular signal, media was removed and cells were washed with 200 mM acetic acid, 500 mM NaCl in PBS for 60 seconds. Cells were further washed with PBS, then fixed and permeabilised with ice-cold methanol. Following fixation, cells were blocked with 2% BSA in PBS (IF block) for 1 hour at room temperature. Primary antibodies were diluted in IF block to the required concentration and incubated overnight at 4 °C. Primary antibody was removed, and cells washed three times with PBS. Secondary Alexa-Fluor conjugated antibodies were diluted in IF block and

incubated on cells for 1 hour at room temperature. Antibody was then removed, and cells washed three times in PBS. Hoechst nuclear stain was diluted in PBS and incubated on cells for 10 minutes, followed by three washes in PBS. Slides were mounted using ProLong Diamond Antifade Mountant (Invitrogen, P36961) and cured overnight. Confocal images were acquired on an SP8 Lightning Confocal microscope (Leica). For quantification of colocalised particles, at least six z-stack images were obtained per condition (25 steps, 0.3 µm step size) and the maximum intensity projection acquired. DAPI counts were acquired and colocalised puncta were quantified in Fiji-ImageJ using the ComDet plugin (*44*). Puncta less than 4 pixels apart and present in both channels were counted as colocalised and divided by cell number determined via Hoechst staining.

Seeding neutralisation in primary neurons

Primary neuron cultures were prepared from P301S-Tau Tg mice in 96-well plates. On DIV 7, recombinant tau assemblies were incubated with Ab or buffer for 1 hour at room temperature at a 1:5 molar ratio (Tau:Ab). Tau:Ab complexes were diluted in MM and added to neurons at a final concentration of 50 nM tau assemblies and 250 nM Ab. 200 nM TAK-243 (MedChemExpress, HY-100487/CS-0019384) or DMSO were diluted in MM and applied to cultures for 1 hour before application of Tau:Ab complexes. The compound remained on the cells for the duration of the assay. At DIV 14, cells were fixed with methanol, stained for immunofluorescence with MAP2 (ab5392, Abcam) and AT8 (MN1020, Thermo Fisher) antibodies and nuclei stained with Hoechst. High content images were taken of stained neurons using a Ti2-E High Content Microscope (Nikon) at 10x magnification. Analysis was completed with NIS Elements version 5.41.02 software (Nikon). AT8 positive puncta were quantified and divided by cell count determined via Hoechst staining. All conditions were normalised to their respective rIgG control.

Seeding assay in HEK293

Seeding assays were carried out largely as described previously (*24*). HEK293 cells expressing P301S tau-venus were plated at 15,000 cells per well in black 96-well plates in 50 µL OptiMEM (Thermo Fisher, 31985070). Tau assemblies were diluted in 50 µL OptiMEM (Thermo Fisher, 31985070) and added to cells with 0.5 µl per well Lipofectamine 2000 (Thermo Fisher, 11668019). After 1 h, 100 µL complete DMEM was added to each well to stop the transfection process. Cells were incubated at 37 °C in an IncuCyte® S3 Live-Cell Analysis System for 48–72 h after addition of assemblies. Tau-venus aggregates were quantified using ComDet plugin in ImageJ.

Production of antibodies

cDNA encoding the constant domains of WT mIgG2a and the variable heavy chains of AP422 were synthesised and subcloned into pLNOH2 vectors (*45*) with ampicillin resistance by GenScript Biotech Corporation. Corresponding murine kappa light chains were synthesised and subcloned into separate pLNOH2 vectors. Effector-silencing mutations corresponding to codons encoding P329G, L234A, L235A of the heavy chain constant domains were introduced by site-specific mutagenesis. Resulting expression vectors were co-transfected into Expi293F™ cells (Thermo Fisher Scientific, A14527) using an ExpiFectamine™ 293 Transfection Kit (Thermo Fisher Scientific, A14524) according to the manufacturer's instructions. Abs were collected as supernatant 6 days post-transfection and purified on a CaptureSelect™ mouse LC-kappa Affinity Matrix (Thermo Fisher Scientific, 191315005). Protein fractions were eluted with 0.1 M glycine-HCl (pH 2.7) and neutralised by adding 1M Tris-HCl (pH 8.0). Eluates were concentrated and buffer-exchanged into PBS on 50K Amicon® Ultra-15 Centrifugal Filter Units (Merck Millipore, UFC905096) followed by size-exclusion chromatography to isolate monomeric fractions using a Superdex™ 200 10/300 GL column (Cytiva) coupled to an Äkta Avant 25 (Cytiva). Eluted monomeric Abs were concentrated on 50K Amicon® Ultra-4 Centrifugal Filter Units (Merck Millipore, UFC810024) and subjected to SDS-PAGE using a Bolt™ 12% Bis-Tris polyacrylamide gel (Thermo Fisher Scientific, NW00125BOX) to evaluate protein integrity. Abs from hybridoma were purified from culture supernatant on Protein G HiTrap HP column (Cytiva) coupled to an Äkta Pure

system (Cytiva). Protein was eluted using 0.1 M glycine (pH 2.7) and neutralised in 1M Tris-HCl (pH 9.0). Antibodies were buffer exchanged to PBS using 12000 MWCO SpectraPor membranes and concentrated on Vivaspin 50,000 MWCO Centrifugal Concentrators (Cole-Parmer). All Abs were snap frozen for storage at -80 °C.

T21 PRYSPRY production

6xHis-tagged human T21 PRYSPRY was expressed in *E. coli* (C41 strain) and purified using nickel affinity chromatography and Size Exclusion Chromatography (SEC). Briefly, cells were grown in 2xTY (supplemented with 0.5% glucose, 2 mM $MgSO_4$ and appropriate antibiotics) at 37 °C for 2–3 h (OD600 around 0.6–1), after which they were induced with 1 mM IPTG and incubated at 18 °C overnight. Cells were pelleted with a Sorvall SLC-6000 compatible centrifuge at 4500× g for 25 min and the pellet snap frozen until processed. The pellet was resuspended in lysis buffer (50 mM Tris pH 8, 1 M NaCl, 10% v/v BugBuster (Merck, Gillingham, UK), 10 mM imidazole, 2 mM DTT and 1 × complete protease inhibitors (Roche, Basel, Switzerland) and sonicated for 15 min total time (10 s on/20 s off) at 70% amplitude. The soluble fraction was recovered by centrifugation at 40,000× g in a JLA25.50 rotor and put through a gravity flow column with 5 mL of NiNTA Agarose (Qiagen). The bound fraction was washed in Buffer B (300 mM NaCl, 50 mM Tris pH 8, 10 mM imidazole and 1 mM DTT) and eluted with Buffer E (300 mM NaCl, 50 mM Tris pH 8, 400 mM imidazole and 1 mM DTT). Fractions containing the protein were pooled, filtered, and separated by SEC using HiLoad 26/600 Superdex 75 pg column (Cytiva, Marlborough, MA, USA) in 150 mM NaCl, 50 mM Tris pH 8 and 1 mM DTT. The appropriate fractions were pooled and concentrated to 10–15 mg/ml.

T21 PRYSPRY fluorescence anisotropy

Recombinant 6xHis human TRIM21 PRYSPRY was labelled using Alexa Fluor 488 Microscale Protein Labeling Kit (A30006), following the manufacturer's instructions. Following labelling, 5nM labelled PRYSPRY was mixed with titrated antibodies in PBS + 0.01% Tween 20 for 20 minutes at RT.

Polarisation signal was read on a BMG Clariostar plate reader (excitation 485 nm, emission filter for channel A 520nm, emission filter for channel B 520nm).

Tau production

The expression and purification of recombinant human 6xHis-0N4R tau bearing the P301S mutation from *E. coli* BL-21 (DE3, Agilent Technologies) was performed as described previously (*27,46*) with small modifications. Bacterial pellets were collected through centrifugation (3300 g, 4 °C, 10 min) and then resuspended in 10 ml/L of culture with buffer A (25 mM HEPES, pH 7.4, 300 mM NaCl, 20 mM Imidazole, 1 mM benzamidine, 1 mM PMSF, 14 mM β-mercaptoethanol, 1% NP-40, 1 x complete EDTA-free protease inhibitors). The resuspended bacteria were lysed on ice using a probe sonicator and boiled for 10 min at 95 °C which denatures the majority of proteins, but not tau. Denatured proteins were pelleted by ultracentrifugation at 100,000 g, 4 °C for 50 min. The clarified supernatant containing 6xHis-tagged monomeric tau P301S was then passed through a HisTrap FF column according to manufacturer instructions (GE Healthcare). Eluted fractions were assessed through SDS-PAGE and total protein staining with Coomassie InstantBlue. Fractions of interest were concentrated using 10 kDa cut-off Amicon Ultra-15 concentrators (Merck Millipore) before loading on a HiLoad 16/600 Superdex 200 (Cytiva) size exclusion chromatography column. To remove the 6xHis tag, tau was subjected to TEV protease following manufacturer's instructions (Sigma-Aldrich, T4455). After incubation, the pooled fractions were loaded onto a second HisTrap HP column to remove protease and the successfully cleaved tau collected. Cleaved tau was concentrated using a 10 kDa cut-off Amicon Ultra-15 concentrator (Merck Millipore) before loading onto a HiLoad 16/600 Superdex 200 (Cytiva) size exclusion chromatography column. All purification was performed on an ÄKTA Pure system (Cytiva). Purified tau was concentrated to at least 3 mg/mL using a 10 kDa cut off AmiconUltra-15 concentrator (Merck Millipore) and snap-frozen in liquid nitrogen for storage at -80 °C in PBS containing 1 mM DTT. Levels of endotoxin were measured using a limulus amebocyte lysate kit and found to be below 0.01 EU/ml at tau concentrations used in experiments.

Tau aggregation

Tau monomer was aggregated as described previously (*27*). Briefly, 60 µM tau monomer was incubated with 20 µM heparin, 2 mM DTT and 1X protease inhibitors in PBS for 24–72 h at 37°C shaking at 250 RPM. Thioflavin T (ThT, Thermo Fisher, T3516) was used to quantify Tau aggregation. Tau assemblies were then diluted in PBS to 20 µM monomer equivalent, snap-frozen in liquid nitrogen and stored at -80 °C.

Tau phosphorylation

Recombinant tau assemblies at 12 mM monomer equivalent were treated with ERK2 (Abcam), which is a confirmed kinase of S422 (*33*). Reactions were performed in the presence of 100 mM ATP and Halt protease inhibitors in TBS at 30 °C overnight. Tau was pelleted by ultracentrifugation at 100,000× *g* at 4 °C for 1 h. ERK2 was removed by aspirating the supernatant, and the p-tau was resuspended in PBS with 1x protease inhibitors and 1x phosphatase inhibitors, to a concentration of 20 µM. Aliquots were stored at -80 °C until required.

Preparation of tau from human brain

Tau filaments were obtained from anonymized postmortem tissue donated by patients to the Cambridge Brain Bank under the ethically approved protocol for "Neurodegeneration Research in Dementia" (REC 16/WA/0240). The 4 donors were a 74 year-old female with clinical and pathologically confirmed diagnosis of corticobasal degeneration; a 85 year-old male with clinical and pathologically confirmed diagnosis of progressive supranuclear palsy; a 79 year-old male with a clinical diagnosis of dementia and pathologically confirmed Alzheimer's disease Braak Stage VI, and a 37 year-old male dying of renal failure secondary to type 1 diabetes and no neuropathology (control). 2 g of cortical grey matter was extracted according to a modified version of the method of *(49)*. Briefly, fresh-frozen cortical gray matter was homogenized in 9 volumes of extraction buffer (10 mm Tris-HCl [pH 7.5], 0.8 m NaCl, 10% sucrose,

1 mm EDTA, 0.1 mm PMSF, 0.1% Sarkosyl, 2 mm imidazole, 1 mm NaV, 1 mm NaF, 2 mm DTT, Complete Ultra EDTA-free protease inhibitor mixture [Roche]) using a VelociRuptor V2 homogenizer and tubes prefilled with 2.8-mm acid-washed stainless steel beads. Homogenate was spun at 10,000 × g for 10 min at 4 °C and filtered through a 50-µm cell strainer. The pellet was re-extracted with a further 4.5 volumes of extraction buffer and homogenized and clarified as above. Filtered supernatants were combined, and Sarkosyl was added to a final concentration of 1% before stirring at 100 rpm for 1 h. Samples were then subjected to ultracentrifugation at 100,000 × g for 75 min at 4 °C. The supernatant was separated from the pellet, and the latter was rinsed with PBS before resuspension and vortexing to break it apart. The resuspended pellet was further diluted in PBS and then centrifuged at 130,000 × g for 1 h at 4 °C. The resulting pellet was resuspended in 100 µl per gram gray matter and broken apart by 16 h of agitation at room temperature and passing through 18-, 23-, and 26-gauge needles. The resuspended pellet was sonicated (Hielsher S26D11X10 Vial-Tweeter Sonotrode at settings A 100%, C 50%, and 200 Ws). The sample was then centrifuged at 100,000 × g for 40 min at 4 °C. The pellet was resuspended again in 50 µl PBS per gram of gray matter and subjected to breaking apart using needles and sonication as above. Finally, the sample was subjected to a clearing spin at 10,000 × g at 4 °C. The concentrated Tau filaments were stored at −80 °C prior to use.

Fc-receptor ELISA

To evaluate FcγR and FcRn binding, Corning 96 well plates (Merck, CLS3922) were coated with titrated amounts (5,000-40 ng/ml for FcγRs and 2,000 – 0.91 ng/ml for FcRn) of Ab variants diluted in 1x Dulbecco′s Phosphate Buffered Saline (PBS; Merck, D8537) at a volume of 100 µl per well. Following overnight incubation at 4 °C, plates were washed with 4 x 250 µl of PBS with 0.05% Tween20 (T; Merck, P1379) and blocked with 250 µl of PBS/T containing 4% nonfat dried milk powder (M; VWR, A0830) at room temperature (RT) for 1 h. Between all subsequent layers, plates were washed as previously described. For FcRn ELISAs, phosphate buffer pH 6.0 or PBS-T pH 7.4 were used as wash buffers. Next, biotinylated recombinant soluble murine FcγRI (Sino Biological, 158-50086-M27H-B-100), FcγR2b

(Sino Biological, 158-50030-M27H-B-100), FcγRIII (Sino Biological, 158-50326-M27H-B-100), FcγRIV (Sino Biological, 158-50036-M27H-B-100) or murine FcRn (Immunitrack, ITF08) was incubated with streptavidin-AP conjugate (Roche, 11089161001) at a 1:1 molar ratio for 20 min at RT. The complexes were diluted in PBS/T/M (FcγR and FcRn ELISAs) or phosphate buffer pH 6.0 (FcRn ELISA) and added to the plate at final concentrations of 0.25 μg/ml FcγRs and 3.36 μg/ml streptavidin-AP or 1 μg/ml murine FcRn and 13.44 μg/ml strepdavidin-AP. After 1 h of incubation on a shaker at RT, FcγR or FcRn binding was visualised by adding 100 μl of 10 μg/ml phosphatase substrate (Sigma-Aldrich, S0942) dissolved in diethanolamine solution (pH 9.8). Absorbance was measured at 405 nm with a Sunrise spectrophotometer (Tecan).

T21 PRYSPRY ELISA

T21 binding was tested by coating Corning 96 well plates (Merck, CLS3922) with titrated amounts (10-0.08 μg/ml) of Ab variants diluted in 1x Dulbecco's Phosphate Buffered Saline (PBS; Merck, D8537) at a volume of 100 μl per well. Following overnight incubation at 4 °C and between all subsequent layers, plates were washed with 4 x 250 μl of PBS with 0.05% Tween20 (T; Merck, P1379). The plates were then blocked with 250 μl of PBS/T containing 4% nonfat dried milk powder (M; VWR, A0830) at room temperature (RT) for 1 h. Next, His-tagged murine T21 PRYSPRY was incubated with Alkaline Phosphatase Anti-6X His tag antibody (Abcam, ab49746) for 20 min at RT, diluted in PBS/T/M and added to the plate at final concentrations of 10 μg/ml PRYSPRY and 0.28 μg/ml Alkaline Phosphatase Anti-6X His tag antibody. After 1 h of incubation on a shaker at RT, PRYSPRY binding was visualised by adding 100 μl of 10 μg/ml Phosphatase substrate (Sigma-Aldrich, S0942) dissolved in diethanolamine solution (pH 9.8). Absorbance was measured at 405 nm with a Sunrise spectrophotometer (Tecan).

Surface plasmon resonance (SPR)

T21 binding kinetics were assessed using a Biacore 3000 instrument. 300 resonance units (RU) of the Ab variants was immobilised on Series S CM5 sensor chips (Cytiva, 29149603) using an Amine Coupling

Kit (Cytiva, BR100050) following the manufacturer's instructions. The remaining surface area of the chip was inactivated by injecting 1M ethanolamine-HCl provided in the Amine Coupling Kit. Next, titrated amounts (1000-15.63 nM) of monomeric His-tagged murine T21 PRYSPRY was injected over the immobilised ligands using a flow rate of 50 µl/minute at 25°C. Phosphate buffer pH 6.0 (67 nM phosphate, 0.1 M NaCl, 0.005% Tween) was used as dilution and running buffer, and HBS-P+ buffer pH 7.4 (0.01M HEPES, 0.15 M NaCl, 0.005% surfactant P20) was used as regeneration buffer. The obtained interaction data was adjusted to a blank sample and the reference flow cell values were subtracted before binding kinetics were determined by using a 1:1 Langmuir binding model in the BIAevaluation software.

Human iPSC cultures

Naïve human iPSCs gene edited to include doxycycline-inducible NGN2 transcription factor (iNeurons(*47*)) were maintained in E8 medium (Stem Cell Technologies) on vitronectin (Thermo Fisher) coated plates. iPSCs were passaged with 4mM EDTA or Accutase (ThermoFisher) and ROCK inhibitor Y-27632 (BD Biosciences) when 70% confluency was reached. Differentiation into cortical neurons was performed according to modified versions of existing experimental protocols (*47*). In brief, iPSCs were differentiated on Geltrex coated plates using DMEM/F-12 media supplemented with non-essential amino acids (NEAA) (1x), P/S (1x), glutamine (Q) (1x), N2 supplement (1x), 50µM 2-Mercaptoethanol and Doxycycline (Dox) (2 µg/ml) for the first two days. For Differentiation from days 3-14, Neurobasal media was supplemented with penicillin-streptomycin (1x), L-Glutamine (1x), B-27 supplement (1x), NT-3 (10 ng/ml), 2-Mercaptoethanol (50 µM), Dox (2 µg/ml) and BDNF (10 ng/ml). Full media changes were performed daily until day 6, after which half-media changes were performed every other day. At day 3, the neurons were dissociated into single cells using Accutase and seeded onto Geltrex coated plates. Cells were seeded into 12-well plates at 1 million cells/well for immunoblotting, or into 96-well plates at 40,000 cells/well for adenovirus neutralisation assays. DIV13 neurons were treated with human IFN-α (Sigma-Aldrich, SRP4596) at 5000 IU/ml for 16h before lysis in appropriate volume of 1x RIPA buffer

(Sigma-Aldrich, R0278). For adenovirus infection experiments, adenovirus type 5 vector expressing eGFP under the human synapsin promoter, Ad-SYN-GFP (Signagen, SL100718) was mixed with anti-hexon antibody expressed as human rh9C12 IgG1, PBS or rh9C12 IgG1-Fc region H433A at 70 µg/mL, and incubated for 1 h to allow binding to reach equilibrium. Complexes were added to DIV14 neurons by dilution at 1:20 into media. After 48h incubation at 37°C, cells were dissociated with Accutase into a single cell suspension. GFP-positive cells were analysed by flow cytometry (CytoFlex).

Immunoblot analysis

Lysates were cleared by centrifugation and resuspended with 4x NuPAGE LDS sample buffer (Thermo Fisher, NP0007) with 2mM β-mercaptoethanol, before boiling for 5 minutes. Samples were subjected to SDS-PAGE using NuPAGE Bis-Tris 4-12% gels (Thermo Fisher, NP0324BOX) and transferred to 0.2 µm PVDF membrane using the Bio-Rad Transblot Turbo Transfer System. The membrane was blocked in 5% milk or 5% normal goat serum (NGS) with 0.2x fish gelatin in TBS-T (0.1% Tween-20 in TBS) for 1 h at room temperature before incubation with primary antibodies directed against human T21 (Santa Cruz Biotechnology, sc-25351), CypB (Santa Cruz Biotechnology, sc-130626), STAT1 (Cell Signalling Technology, 9172) and PSD-95 (Millipore, MABN68), phospho-tau ((Ser202, Thr205), AT8, Thermo Fisher, MN1020), phospho-tau ((Thr212, Ser214), AT100, Thermo Fisher, MN1060), phosho-tau ((Ser422), Abcam, ab79415), tau monoclonal antibody (HT7, Thermo Fisher Scientific, MN1000). Membranes were incubated in primary antibody overnight at 4 ºC and following repeated washes with TBS-T, were incubated with secondary HRP/Alexa-Fluor/DyLight conjugated antibodies for 1 h at room temperature. Membranes were washed with TBS-T and incubated with HRP substrate where appropriate (Millipore, WBKLS0500) before imaging with the ChemiDoc system (BioRad).

Dot blot

Samples were transferred onto a 0.2-µm nitrocellulose membrane (Roti-NC transfer membrane, Carl Roth) using the Bio-Dot apparatus (Bio-Rad). Subsequently, the membrane was immunoblotted with the

indicated antibody before probing with secondary antibodies conjugated to Alexa488/555/647 fluorophores and further processed as immunoblots above.

Epitope mapping

Custom membranes with overlapping 15mer peptides in 3 amino acid increments of 0N4R tau were purchased from Peptides & Elephants. Membranes were activated by incubation with 100% methanol for 10 minutes with shaking. Activated membranes were washed three times with TBS for 5 minutes. Membranes were blocked in 5% sucrose, 4% milk in TBS-T (0.05% Tween-20 in TBS) (blocking buffer) for 2 hours, before being washed once for 5 minutes with TBS-T. Primary antibodies were diluted in blocking buffer and incubated with the membranes overnight at 4°C. The following morning, membranes were washed three times with TBS-T for 5 minutes. Secondary fluorescent Dylight antibodies were diluted in blocking buffer and incubated with the membranes for 2 hours at room temperature. Membranes were washed three times with TBS-T for 5 minutes, followed by one wash with TBS. Images were taken with the ChemiDoc system (BioRad). The intensity of each dot was determined using Fiji-ImageJ Gel Analyzer. Results were plotted against the amino acid start site of each 15mer peptide, relative to the brightest dot on the membrane. Membranes were stripped with 8 M urea, 1% SDS in TBS overnight, at 37°C whilst shaking and reused as necessary.

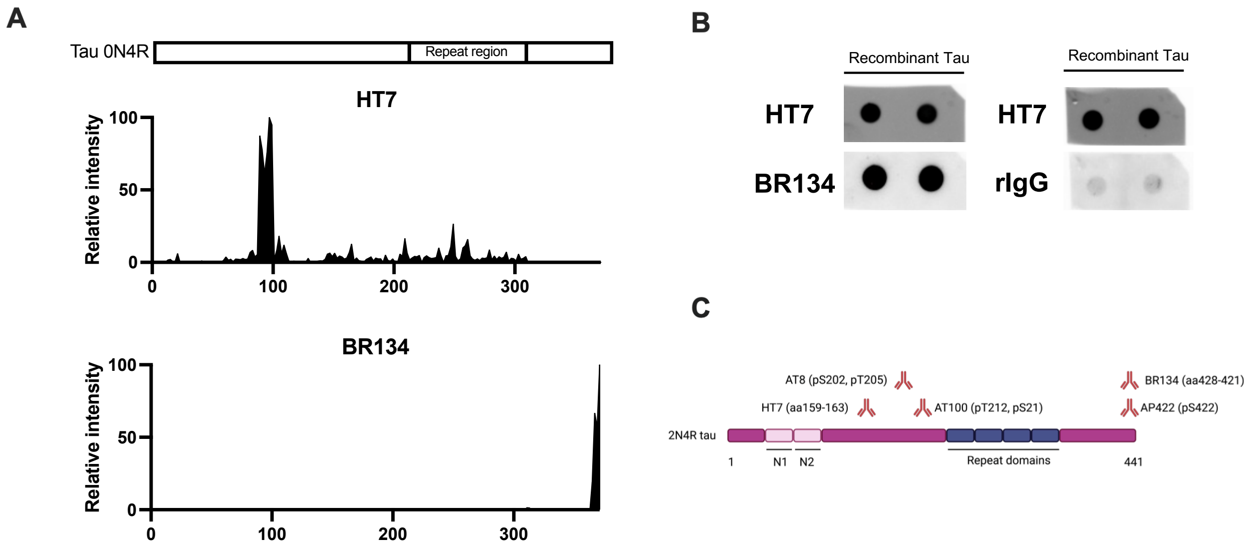

Fig. S1.

BR134 antibody characterisation. A) Epitope mapping of the N-terminal specific monoclonal antibody HT7 and the C-terminal polyclonal BR134 using against overlapping peptides of 0N4R tau. Amino acid refers to start site of 15mer peptides, numbering specific 0N4R tau. B) Dot blot against immobilised recombinant tau using HT7, BR134, or an isotype control antibody. C) Schematic of anti-tau antibodies used in this study and the location of their epitopes on 2N4R tau. aa, amino acid according to human 2N4R tau numbering.

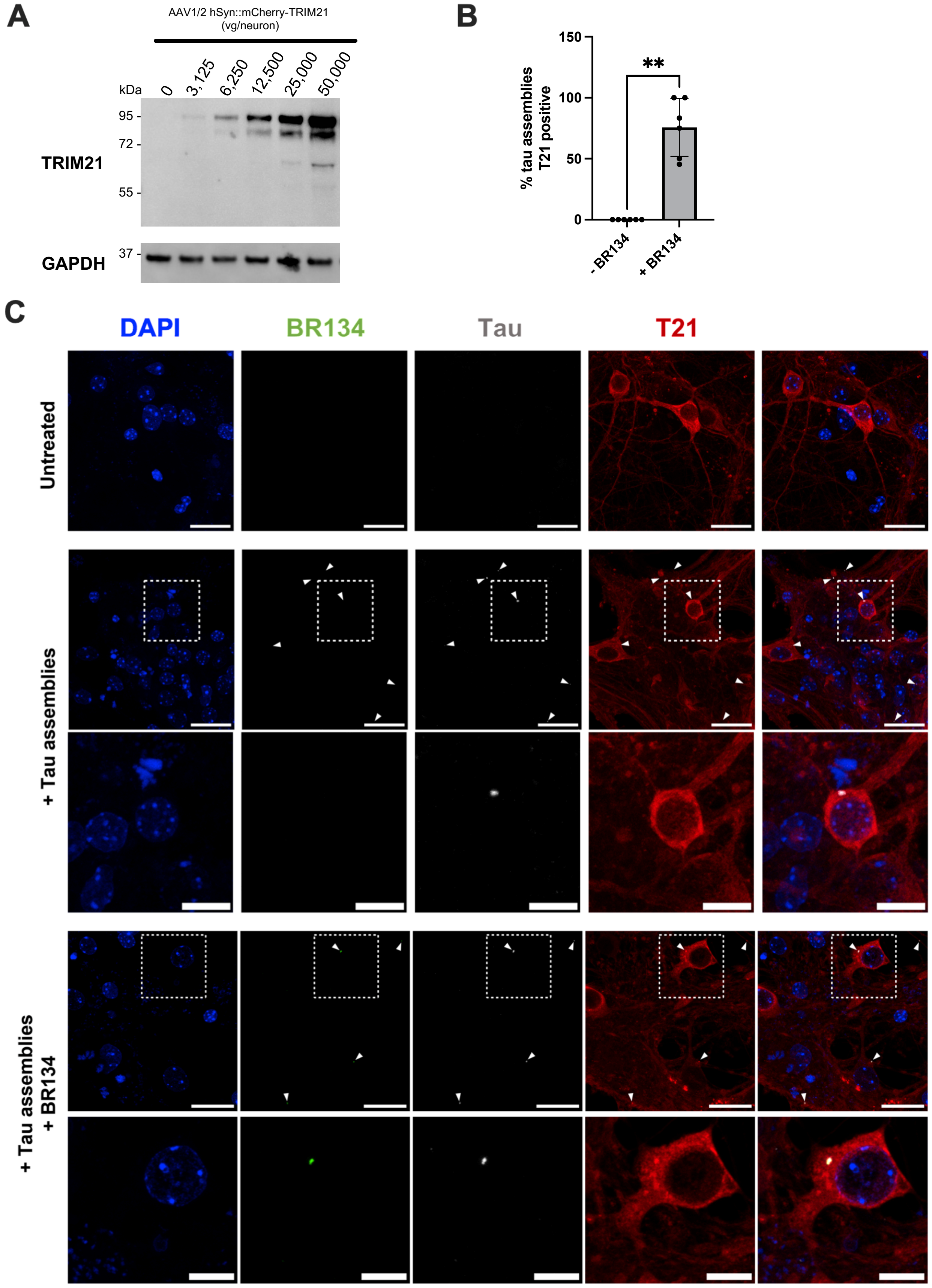
A
AAV1/2 hSyn::mCherry-TRIM21
(vg/neuron)
0
3,125
6,250
12,500
25,000
50,000
kDa
95
72
55
37
TRIM21
GAPDH
B
% tau assemblies
T21 positive
150
100
50
0
**
- BR134
+ BR134
C
DAPI
BR134
Tau
T21
Untreated
+ Tau assemblies
+ Tau assemblies
+ BR134

Fig. S2.

Cytosolic entry of tau assemblies in complex with Abs. A) Primary neurons prepared from T21$^{-/-}$ mice were treated with indicated amounts of chimeric AAV1/2 particles encoding mCherry-tagged mouse *Trim21* under the hSyn promoter. Immunoblot for TRIM21 and loading control GAPDH. vg, viral genomes. B) Percent of intracellular tau assemblies positive for mCh-T21 puncta in the presence or absence of BR134. C) Control images for Fig 1A demonstrating the absence of mCh-T21 foci at site of intracellular tau assemblies when antibody is absent. AAV-treated neurons were challenged with tau assemblies with or without antibody BR134 and fixed for immunofluorescence analysis. Scale bar 25 µm, inset scale bar 10 µm. B) Mean +/- sd; Mann-Whitney test, **, P<0.01.

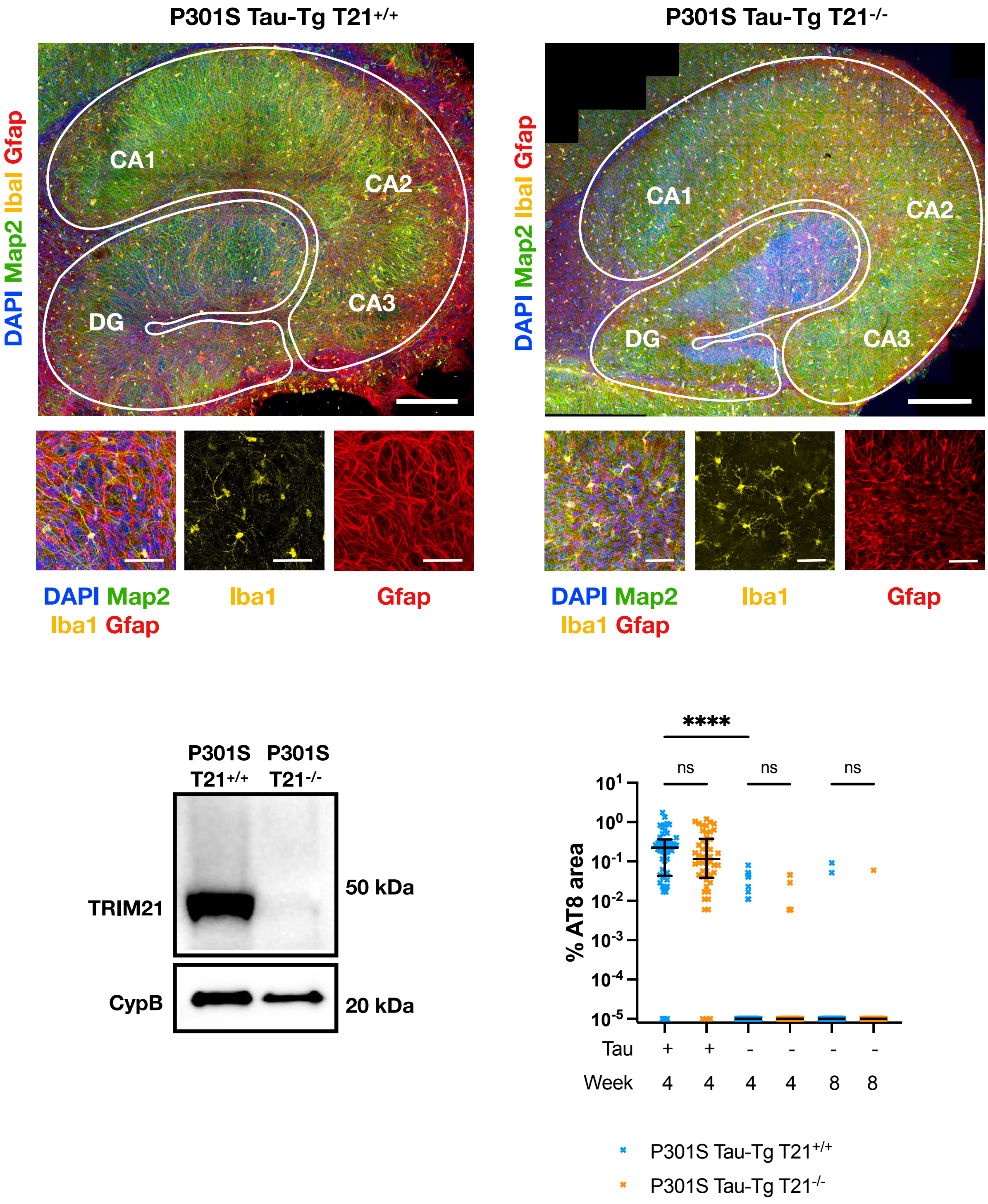

Fig. S3.

Characterisation of organotypic hippocampal slice cultures. A) Tiled fluorescence microscope images of OHSCs prepared from P301S Tau-Tg T21$^{+/+}$ and P301S Tau-Tg T21$^{-/-}$ mouse strains immunostained for Map2 (neuronal marker), Iba1 (microglial marker) and Gfap (astrocyte marker). B) Immunoblot demonstrating expression of TRIM21 in OHSCs prepared from P301S Tau-Tg mice that were T21$^{+/+}$ or

$T21^{-/-}$. CypB, cyclophilin B loading control. C) Levels of AT8 reactivity following treatment with recombinant tau assemblies, or untreated slices over 4-8 weeks. Median with interquartile range of images from multiple slices from N=3 mice. Kruskal-Wallace test by ranks with Dunns multiple comparisons test ****, P<0.0001. Scale bar 250 µm, 50 µm inset.

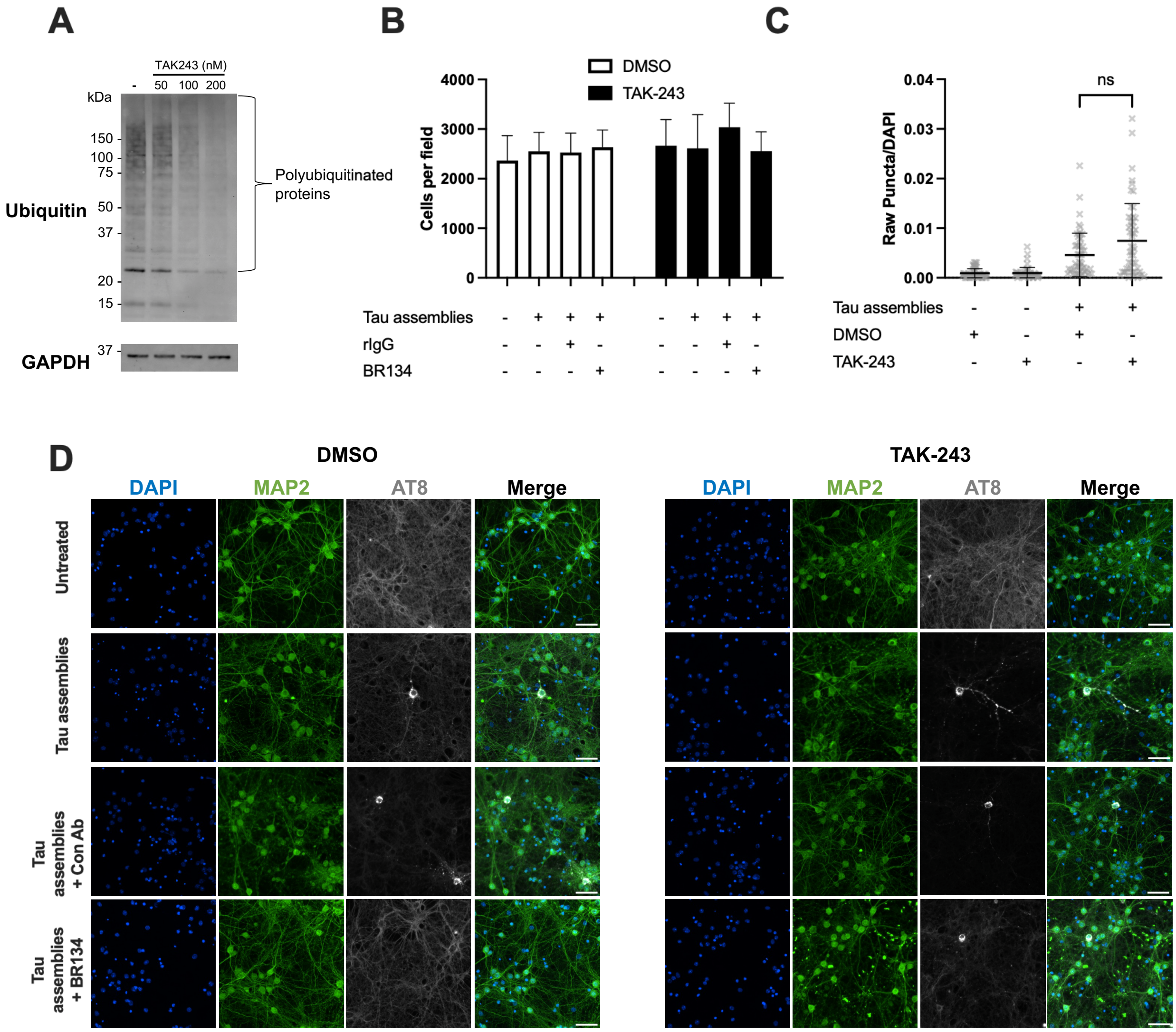

Fig. S4

Neutralisation of tau seeding is abrogated by the ubiquitination inhibitor TAK-243. A) Lysates prepared from primary mouse neurons transgenic for human P301S tau, either untreated or treated with the indicated concentration of the E1 ubiquitin activating enzyme inhibitor TAK-243, were analysed by immunoblotting for levels of polyubiquitinated proteins. B) Immunofluorescence analysis of fixed neurons stained with the DNA stain Hoechst 33342, to quantify number of cells across different treatment groups, mean +/- sd. C) Levels of AT8 positive puncta in primary mouse neurons transgenic for human P301S tau challenged with recombinant tau assemblies in the presence of either DMSO or TAK-243 at 200 mM. Mean +/- sd with Mann-Whitney test. D) Representative immunofluorescence images for AT8-reactive tau structures in primary mouse neurons prepared from P301S Tau-Tg mice challenged with tau assemblies in the presence of DMSO or TAK-243 that were untreated or incubated with BR134 or control rabbit IgG. Scale bar 50 µm. ns, not significant.

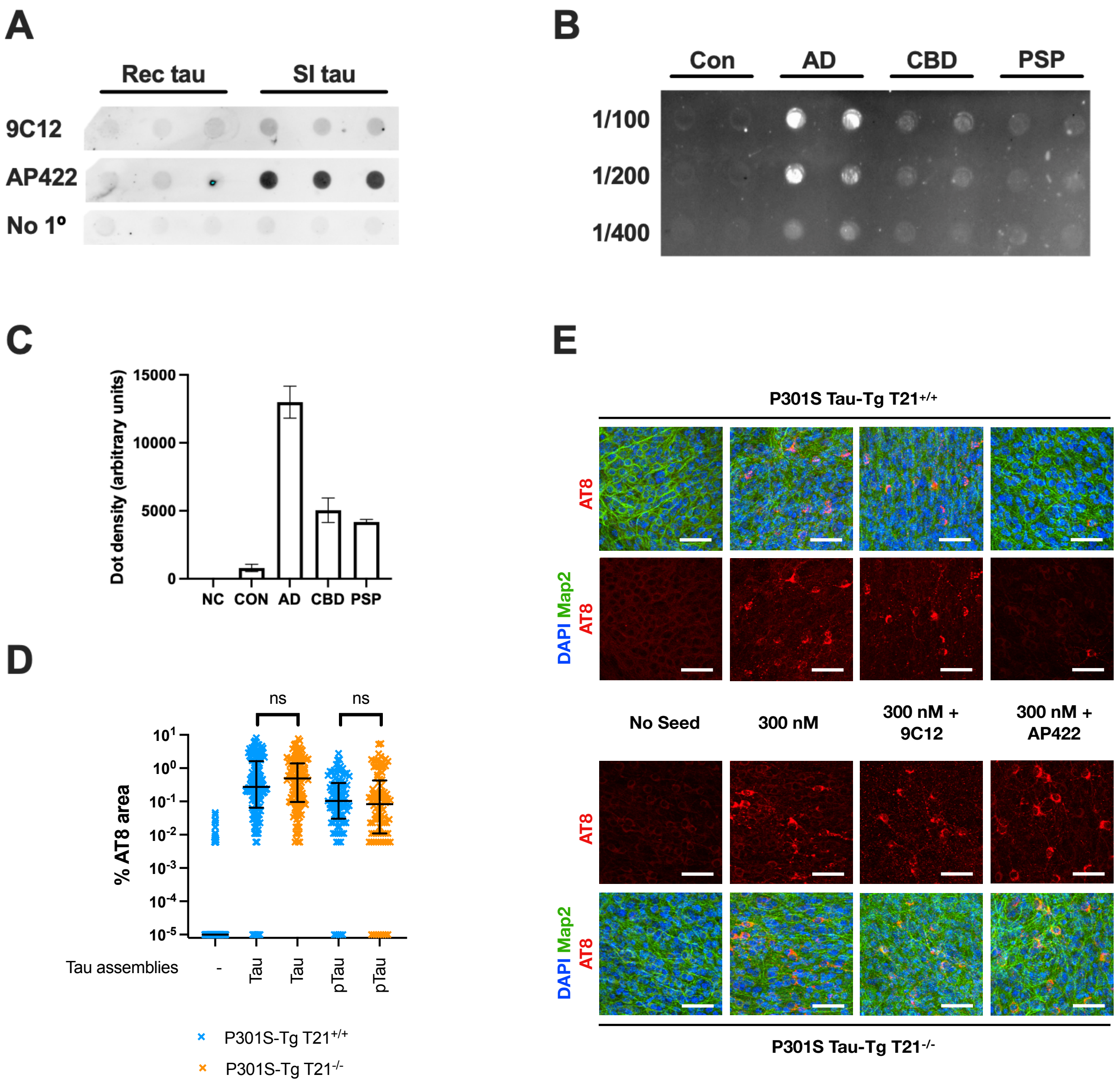

Fig. S5

Characterisation of AP422 antibody. A) Dot blot using phospho-tau antibody AP422 or anti-adenovirus antibody 9C12 against immobilised recombinant (rec) tau, which is not phosphorylated, and sarkosyl insoluble (SI) tau prepared from P301S tau transgenic mice, which is hyperphosphorylated. B) Dot blot showing levels of AP422 reactivity against SI tau prepared from brain tissue from histologically confirmed Alzheimer's disease (AD), corticobasal degeneration (CBD) and progressive supranuclear palsy (PSP) or an individual with no observable tau pathology (Con). A dilution series of SI fraction corresponding to equal volumes of brain tissue was loaded for each patient. C) Graph of relative reactivity of AP422 to SI preparations, as measured by dot blot in (B). Mean +/- sd. D) Levels of AT8 reactivity in OHSCs following treatment with recombinant tau assemblies that were untreated (Tau) or treated (pTau) with kinases. Median +/- interquartile range; Kruskal-Wallis test with Dunn's multiple

comparisons. E) Representative immunofluorescence images for AT8-reactive tau structures in OHSCs from $T21^{+/+}$ and $T21^{-/-}$ backgrounds challenged with phospho-tau assemblies that were untreated or incubated with the indicated Ab. Scale bar 50 µm; ns, not significant.

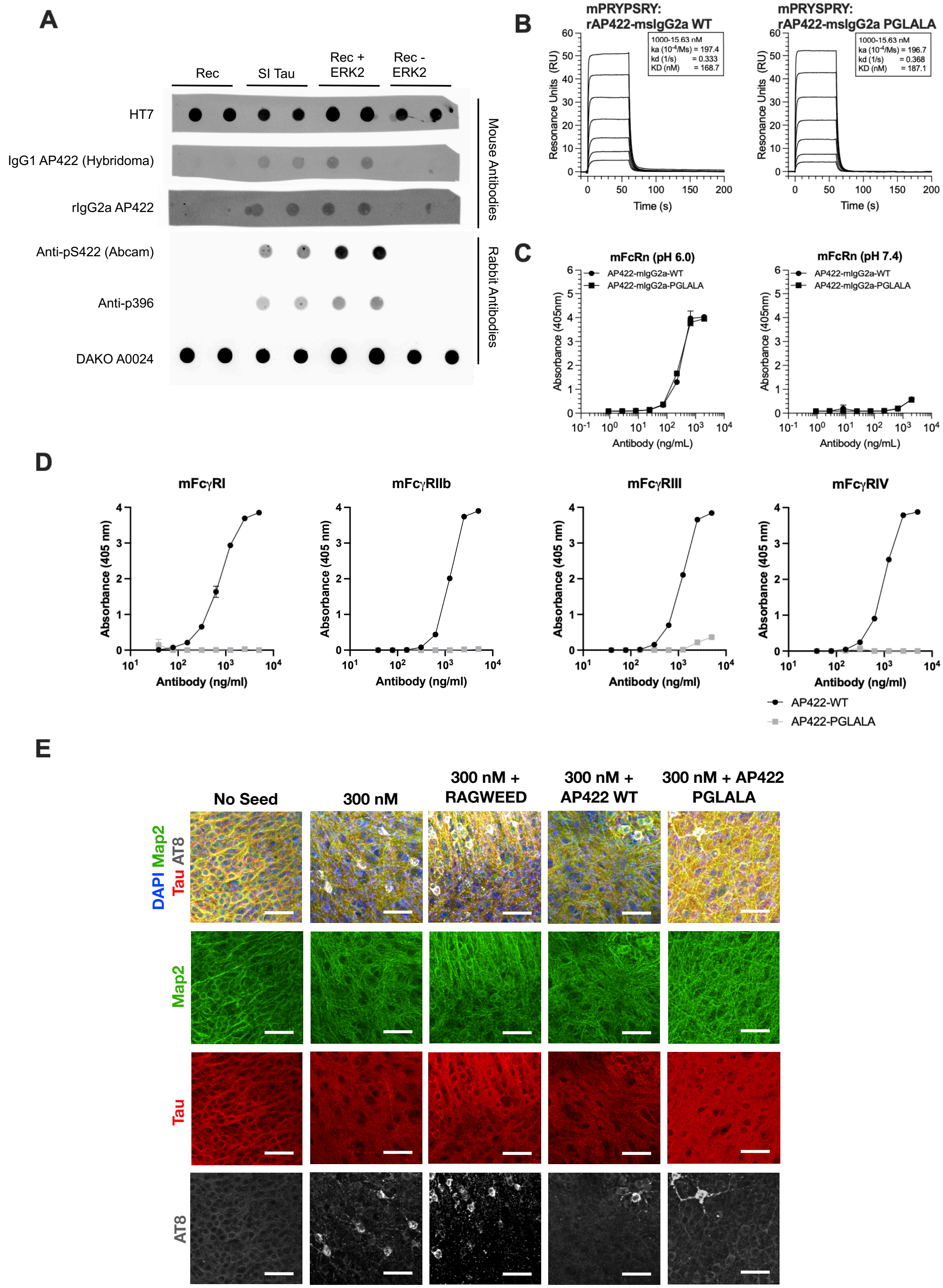
A
Rec
SI Tau
Rec + ERK2
Rec - ERK2
HT7
IgG1 AP422 (Hybridoma)
rIgG2a AP422
Anti-pS422 (Abcam)
Anti-p396
DAKO A0024
Mouse Antibodies
Rabbit Antibodies
B
mPRYPSRY: rAP422-msIgG2a WT
mPRYSPRY: rAP422-msIgG2a PGLALA
Resonance Units (RU)
Time (s)
C
mFcRn (pH 6.0)
mFcRn (pH 7.4)
AP422-mIgG2a-WT
AP422-mIgG2a-PGLALA
Absorbance (405nm)
Antibody (ng/mL)
D
mFcγRI
mFcγRIIb
mFcγRIII
mFcγRIV
Absorbance (405 nm)
Antibody (ng/ml)
AP422-WT
AP422-PGLALA
E
No Seed
300 nM
300 nM + RAGWEED
300 nM + AP422 WT
300 nM + AP422 PGLALA
DAPI Map2 Tau AT8
Map2
Tau
AT8

Fig. S6

Characterisation of recombinant AP422. A) Dot blot against immobilised recombinant tau assemblies (rec), P301S Tau-Tg mouse brain sarkosyl insoluble tau (SI) or recombinant tau assemblies that were untreated or treated with ERK2 kinase. Membranes were probed using either HT7 (total tau); hybridoma origin AP422; recombinant AP422 expressed at mouse IgG2a; commercial rabbit phospho-tau specific Abs, anti-pS422 and anti-pS396, or the tau repeat region-specific Ab, DAKO A0024. B) PRYSPRY:Ab binding kinetics measured by surface plasmon resonance of recombinant AP422 made as mouse IgG2a and expressed as wildtype or as an Fc-engineered version containing the PGLALA substitutions. C) ELISA measurement of recombinant AP422 IgG2a binding to mouse FcRn at the indicated pH. Titrated amounts of the antibodies were coated in ELISA wells followed by addition of the recombinant soluble form of FcRn that was site-specifically biotinylated. Mean +/- sd from N=2 repeats. D) ELISA measurement of recombinant AP422 IgG2a binding to mouse FcγRs. Titrated amounts of the antibodies were coated in ELISA wells followed by addition of recombinant soluble forms of the following receptors that were site-specifically biotinylated. mFcγRI (Fcgr1, CD64); mFcγRIIb (Fcgr2, CD32); mFcγRIII (Fcgr3, CD16); mFcγRIV (Fcgr4, CD16.2). Bound receptors were detected using alkaline phosphate-conjugated streptavidin. Mean +/- sd from N=2 repeats. E) Representative immunofluorescence images for AT8-reactive tau structures in OHSCs from P301S Tau-Tg mice challenged with phospho-tau assemblies that were untreated or incubated with the indicated recombinant Ab. Scale bar 50 μm.

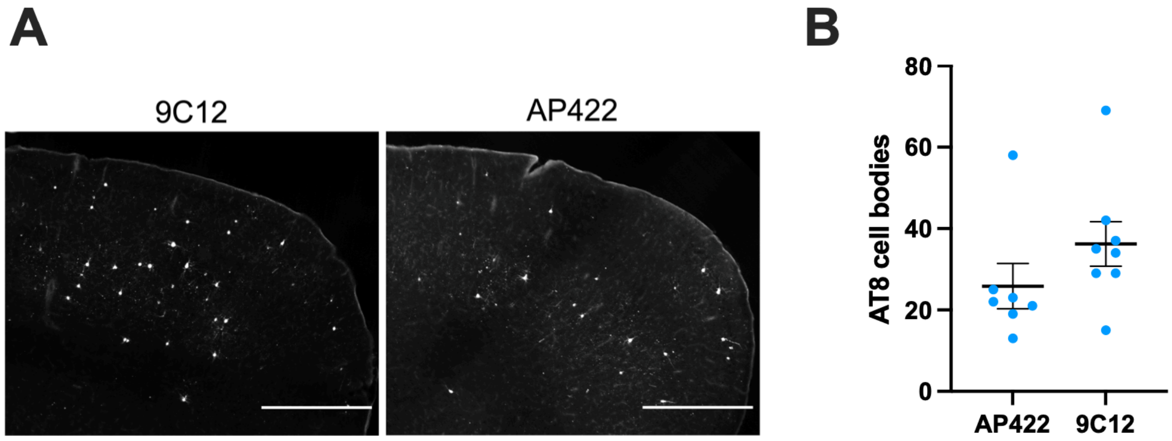

Fig. S7

Immunohistochemistry analysis of fixed brain samples. A) Representative images of frontal cortex slices from P301S Tau-Tg mice stained with AT8. Images were selected using median sarkosyl insoluble HT7 by immunoblot. B) Quantification of AT8 positive cell bodies in the cerebral cortex. Points represent individual mice; N=7 AP422, N=8 9C12; Mann-Whitney test, P=0.067 and P<0.05 when one high outlier animals is excluded. Scale bar, 100 µm.
